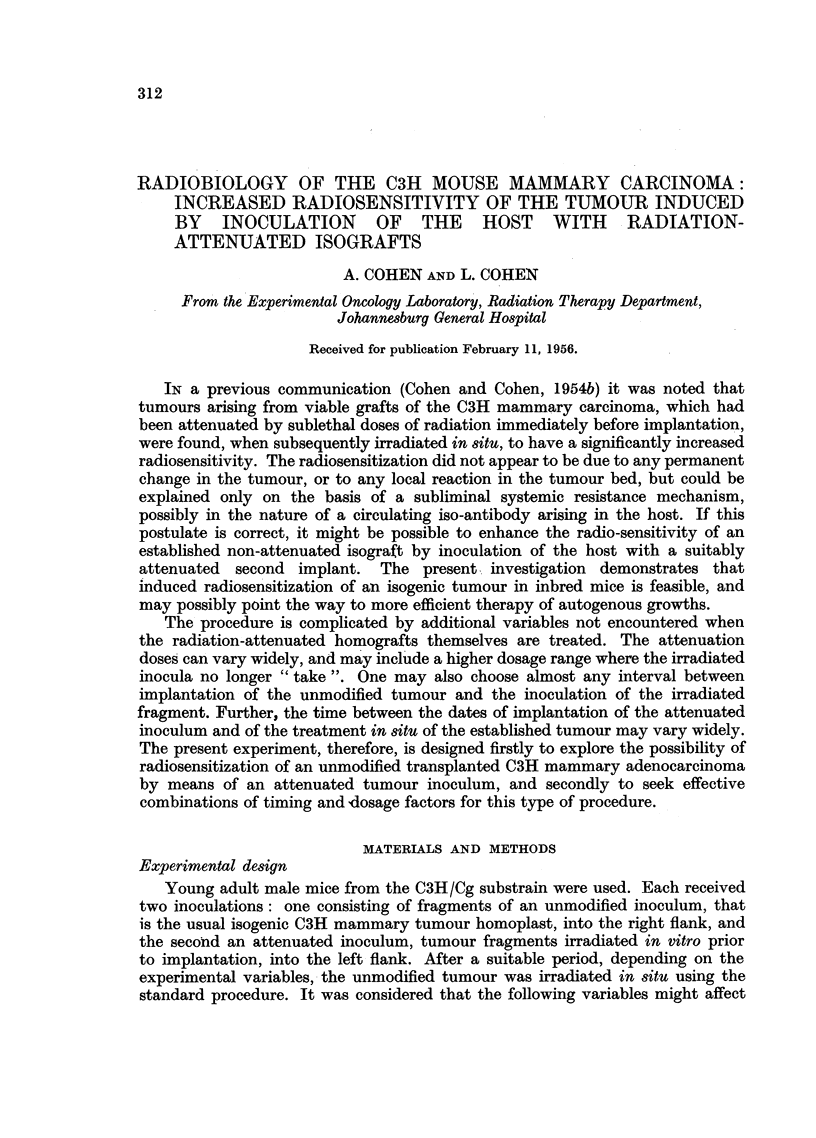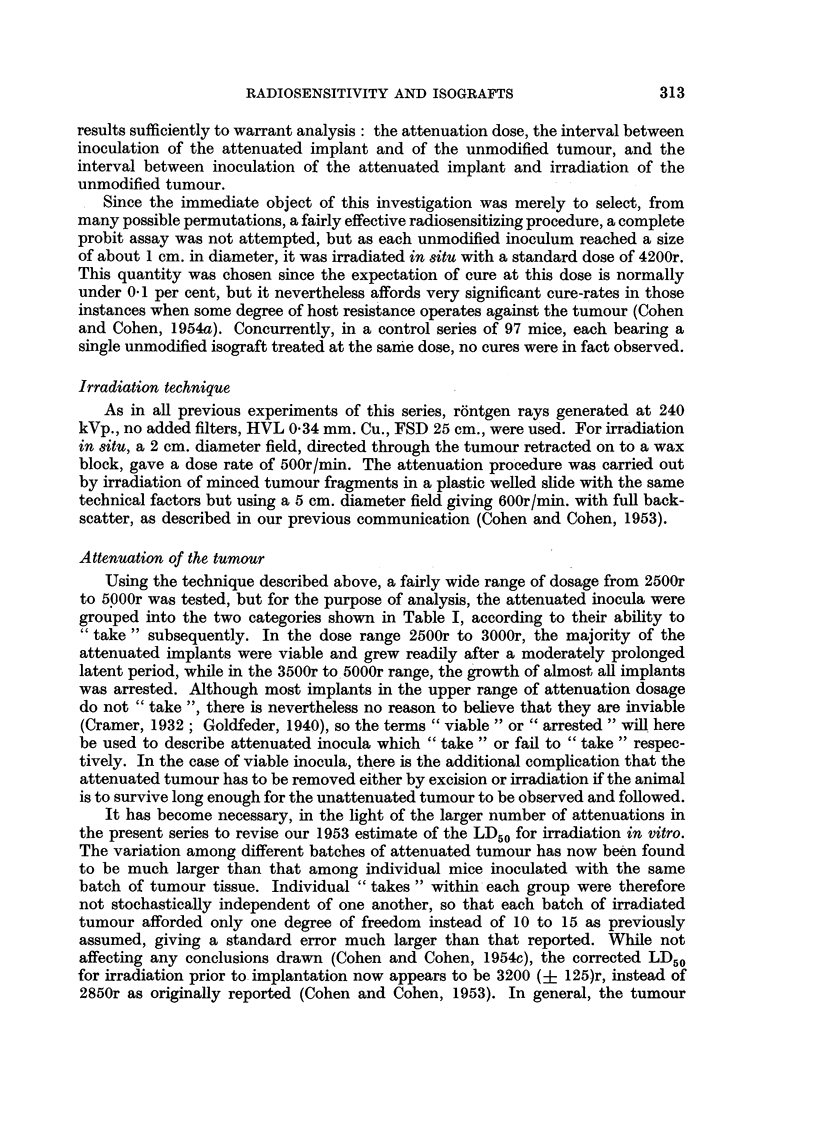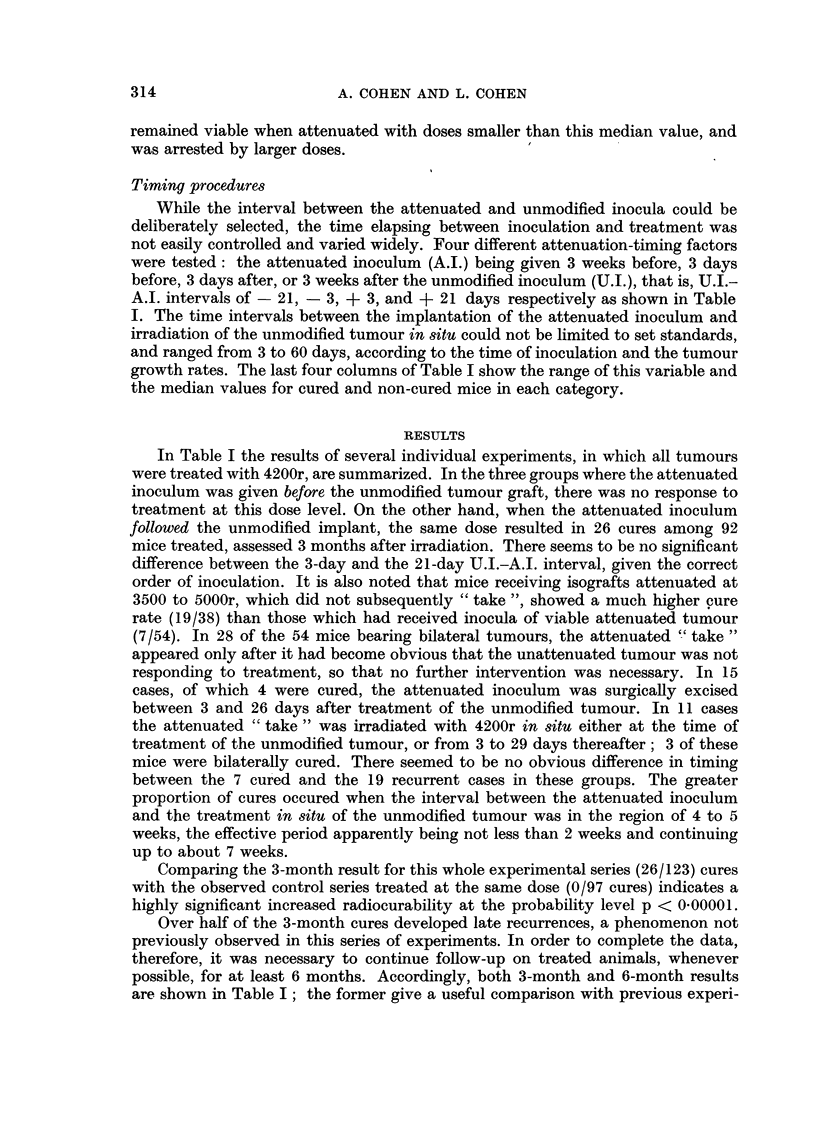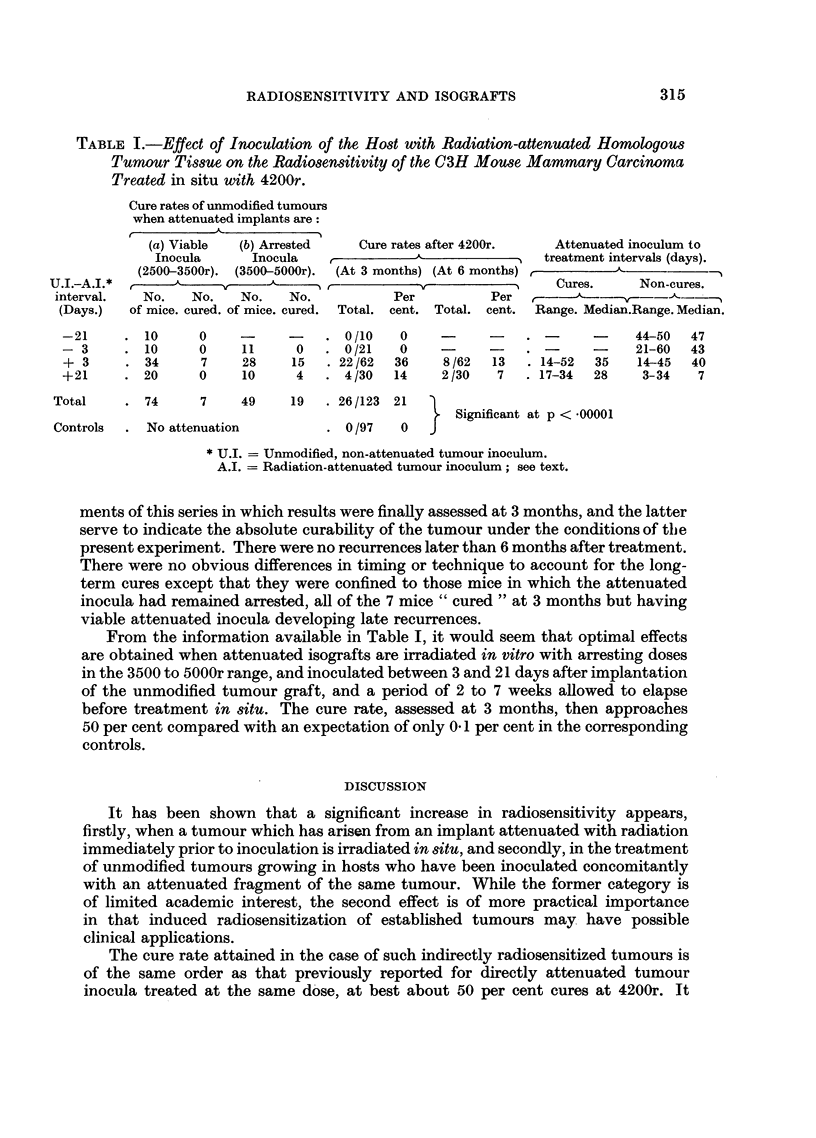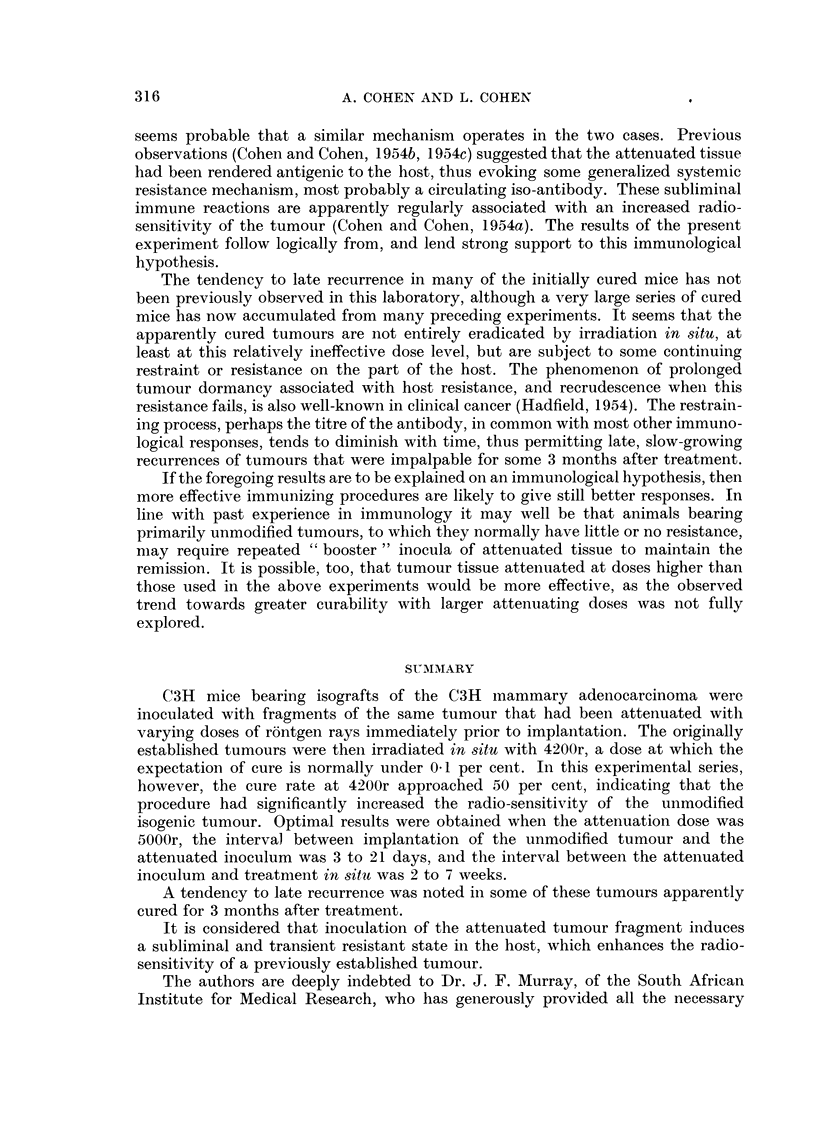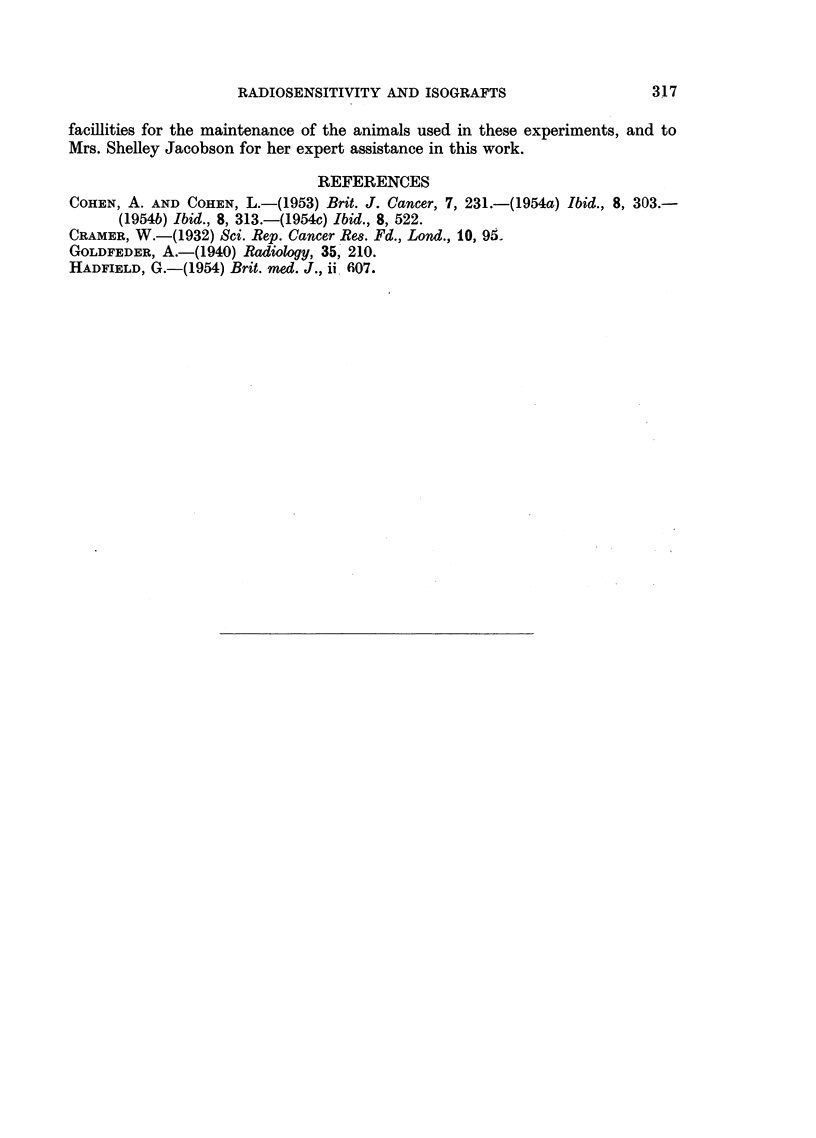# Radiobiology of the C3H Mouse Mammary Carcinoma: Increased Radiosensitivity of the Tumour Induced by Inoculation of the Host with Radiation-Attenuated Isografts

**DOI:** 10.1038/bjc.1956.36

**Published:** 1956-06

**Authors:** A. Cohen, L. Cohen


### RADIOBIOLOGY OF THE C3H MOUSE MAMMARY CARCINOMA: INCREASED RADIOSENSITIVITY OF THE TUMOUR INDUCED BY INOCULATION OF THE HOST WITH RADIATION-ATTENUATED ISOGRAFTS

A. COHEN AND L. COHEN

*From the Experimental Oncology Laboratory, Radiation Therapy Department, Johannesburg General Hospital*

Received for publication February 11, 1956.

IN a previous communication (Cohen and Cohen, 1954*b*) it was noted that tumours arising from viable grafts of the C3H mammary carcinoma, which had been attenuated by sublethal doses of radiation immediately before implantation, were found, when subsequently irradiated *in situ*, to have a significantly increased radiosensitivity. The radiosensitization did not appear to be due to any permanent change in the tumour, or to any local reaction in the tumour bed, but could be explained only on the basis of a subliminal systemic resistance mechanism, possibly in the nature of a circulating iso-antibody arising in the host. If this postulate is correct, it might be possible to enhance the radio-sensitivity of an established non-attenuated isograft by inoculation of the host with a suitably attenuated second implant. The present investigation demonstrates that induced radiosensitization of an isogenic tumour in inbred mice is feasible, and may possibly point the way to more efficient therapy of autogenous growths.

The procedure is complicated by additional variables not encountered when the radiation-attenuated homografts themselves are treated. The attenuation doses can vary widely, and may include a higher dosage range where the irradiated inocula no longer " take ". One may also choose almost any interval between implantation of the unmodified tumour and the inoculation of the irradiated fragment. Further, the time between the dates of implantation of the attenuated inoculum and of the treatment *in situ* of the established tumour may vary widely. The present experiment, therefore, is designed firstly to explore the possibility of radiosensitization of an unmodified transplanted C3H mammary adenocarcinoma by means of an attenuated tumour inoculum, and secondly to seek effective combinations of timing and dosage factors for this type of procedure.

### MATERIALS AND METHODS

### *Experimental design*

Young adult male mice from the C3H/Cg substrain were used. Each received two inoculations: one consisting of fragments of an unmodified inoculum, that is the usual isogenic C3H mammary tumour homoplast, into the right flank, and the second an attenuated inoculum, tumour fragments irradiated *in vitro* prior to implantation, into the left flank. After a suitable period, depending on the experimental variables, the unmodified tumour was irradiated *in situ* using the standard procedure. It was considered that the following variables might affect results sufficiently to warrant analysis : the attenuation dose, the interval between inoculation of the attenuated implant and of the unmodified tumour, and the interval between inoculation of the attenuated implant and irradiation of the unmodified tumour.

Since the immediate object of this investigation was merely to select, from many possible permutations, a fairly effective radiosensitizing procedure, a complete probit assay was not attempted, but as each unmodified inoculum reached a size of about 1 cm. in diameter, it was irradiated *in situ* with a standard dose of 4200r. This quantity was chosen since the expectation of cure at this dose is normally under 0·1 per cent, but it nevertheless affords very significant cure-rates in those instances when some degree of host resistance operates against the tumour (Cohen and Cohen, 1954*a*). Concurrently, in a control series of 97 mice, each bearing a single unmodified isograft treated at the same dose, no cures were in fact observed.

*Irradiation technique*

As in all previous experiments of this series, röntgen rays generated at 240 kVp., no added filters, HVL 0·34 mm. Cu., FSD 25 cm., were used. For irradiation *in situ*, a 2 cm. diameter field, directed through the tumour retracted on to a wax block, gave a dose rate of 500r/min. The attenuation procedure was carried out by irradiation of minced tumour fragments in a plastic welled slide with the same technical factors but using a 5 cm. diameter field giving 600r/min. with full back-scatter, as described in our previous communication (Cohen and Cohen, 1953).

*Attenuation of the tumour*

Using the technique described above, a fairly wide range of dosage from 2500r to 5000r was tested, but for the purpose of analysis, the attenuated inocula were grouped into the two categories shown in Table I, according to their ability to " take " subsequently. In the dose range 2500r to 3000r, the majority of the attenuated implants were viable and grew readily after a moderately prolonged latent period, while in the 3500r to 5000r range, the growth of almost all implants was arrested. Although most implants in the upper range of attenuation dosage do not " take ", there is nevertheless no reason to believe that they are inviable (Cramer, 1932 ; Goldfeder, 1940), so the terms " viable " or " arrested " will here be used to describe attenuated inocula which " take " or fail to " take " respectively. In the case of viable inocula, there is the additional complication that the attenuated tumour has to be removed either by excision or irradiation if the animal is to survive long enough for the unattenuated tumour to be observed and followed.

It has become necessary, in the light of the larger number of attenuations in the present series to revise our 1953 estimate of the $LD_{50}$ for irradiation *in vitro*. The variation among different batches of attenuated tumour has now been found to be much larger than that among individual mice inoculated with the same batch of tumour tissue. Individual " takes " within each group were therefore not stochastically independent of one another, so that each batch of irradiated tumour afforded only one degree of freedom instead of 10 to 15 as previously assumed, giving a standard error much larger than that reported. While not affecting any conclusions drawn (Cohen and Cohen, 1954*c*), the corrected $LD_{50}$ for irradiation prior to implantation now appears to be 3200 ($\pm$ 125)r, instead of 2850r as originally reported (Cohen and Cohen, 1953). In general, the tumour remained viable when attenuated with doses smaller than this median value, and was arrested by larger doses.

*Timing procedures*

While the interval between the attenuated and unmodified inocula could be deliberately selected, the time elapsing between inoculation and treatment was not easily controlled and varied widely. Four different attenuation-timing factors were tested: the attenuated inoculum (A.I.) being given 3 weeks before, 3 days before, 3 days after, or 3 weeks after the unmodified inoculum (U.I.), that is, U.I.–A.I. intervals of − 21, − 3, + 3, and + 21 days respectively as shown in Table I. The time intervals between the implantation of the attenuated inoculum and irradiation of the unmodified tumour *in situ* could not be limited to set standards, and ranged from 3 to 60 days, according to the time of inoculation and the tumour growth rates. The last four columns of Table I show the range of this variable and the median values for cured and non-cured mice in each category.

### RESULTS

In Table I the results of several individual experiments, in which all tumours were treated with 4200r, are summarized. In the three groups where the attenuated inoculum was given *before* the unmodified tumour graft, there was no response to treatment at this dose level. On the other hand, when the attenuated inoculum *followed* the unmodified implant, the same dose resulted in 26 cures among 92 mice treated, assessed 3 months after irradiation. There seems to be no significant difference between the 3-day and the 21-day U.I.–A.I. interval, given the correct order of inoculation. It is also noted that mice receiving isografts attenuated at 3500 to 5000r, which did not subsequently " take ", showed a much higher cure rate (19/38) than those which had received inocula of viable attenuated tumour (7/54). In 28 of the 54 mice bearing bilateral tumours, the attenuated " take " appeared only after it had become obvious that the unattenuated tumour was not responding to treatment, so that no further intervention was necessary. In 15 cases, of which 4 were cured, the attenuated inoculum was surgically excised between 3 and 26 days after treatment of the unmodified tumour. In 11 cases the attenuated " take " was irradiated with 4200r *in situ* either at the time of treatment of the unmodified tumour, or from 3 to 29 days thereafter; 3 of these mice were bilaterally cured. There seemed to be no obvious difference in timing between the 7 cured and the 19 recurrent cases in these groups. The greater proportion of cures occured when the interval between the attenuated inoculum and the treatment *in situ* of the unmodified tumour was in the region of 4 to 5 weeks, the effective period apparently being not less than 2 weeks and continuing up to about 7 weeks.

Comparing the 3-month result for this whole experimental series (26/123) cures with the observed control series treated at the same dose (0/97 cures) indicates a highly significant increased radiocurability at the probability level $p < 0{\cdot}00001$.

Over half of the 3-month cures developed late recurrences, a phenomenon not previously observed in this series of experiments. In order to complete the data, therefore, it was necessary to continue follow-up on treated animals, whenever possible, for at least 6 months. Accordingly, both 3-month and 6-month results are shown in Table I; the former give a useful comparison with previous experi-

TABLE I.—*Effect of Inoculation of the Host with Radiation-attenuated Homologous Tumour Tissue on the Radiosensitivity of the C3H Mouse Mammary Carcinoma Treated* in situ *with* 4200r.

| U.I.–A.I.* interval. (Days.) | Cure rates of unmodified tumours when attenuated implants are : (a) Viable Inocula (2500–3500r). No. of mice. | No. cured. | (b) Arrested Inocula (3500–5000r). No. of mice. | No. cured. | Cure rates after 4200r. (At 3 months) Total. | Per cent. | (At 6 months) Total. | Per cent. | Attenuated inoculum to treatment intervals (days). Cures. Range. | Median. | Non-cures. Range. | Median. |
|---|---|---|---|---|---|---|---|---|---|---|---|---|
| −21 | 10 | 0 | — | — | 0/10 | 0 | — | — | — | — | 44–50 | 47 |
| − 3 | 10 | 0 | 11 | 0 | 0/21 | 0 | — | — | — | — | 21–60 | 43 |
| + 3 | 34 | 7 | 28 | 15 | 22/62 | 36 | 8/62 | 13 | 14–52 | 35 | 14–45 | 40 |
| +21 | 20 | 0 | 10 | 4 | 4/30 | 14 | 2/30 | 7 | 17–34 | 28 | 3–34 | 7 |
| Total | 74 | 7 | 49 | 19 | 26/123 | 21 | Significant at $p < \cdot 00001$ | | | | | |
| Controls | No attenuation | | | | 0/97 | 0 | | | | | | |

* U.I. = Unmodified, non-attenuated tumour inoculum.
A.I. = Radiation-attenuated tumour inoculum ; see text.

ments of this series in which results were finally assessed at 3 months, and the latter serve to indicate the absolute curability of the tumour under the conditions of the present experiment. There were no recurrences later than 6 months after treatment. There were no obvious differences in timing or technique to account for the long-term cures except that they were confined to those mice in which the attenuated inocula had remained arrested, all of the 7 mice " cured " at 3 months but having viable attenuated inocula developing late recurrences.

From the information available in Table I, it would seem that optimal effects are obtained when attenuated isografts are irradiated *in vitro* with arresting doses in the 3500 to 5000r range, and inoculated between 3 and 21 days after implantation of the unmodified tumour graft, and a period of 2 to 7 weeks allowed to elapse before treatment *in situ*. The cure rate, assessed at 3 months, then approaches 50 per cent compared with an expectation of only 0·1 per cent in the corresponding controls.

### DISCUSSION

It has been shown that a significant increase in radiosensitivity appears, firstly, when a tumour which has arisen from an implant attenuated with radiation immediately prior to inoculation is irradiated *in situ*, and secondly, in the treatment of unmodified tumours growing in hosts who have been inoculated concomitantly with an attenuated fragment of the same tumour. While the former category is of limited academic interest, the second effect is of more practical importance in that induced radiosensitization of established tumours may have possible clinical applications.

The cure rate attained in the case of such indirectly radiosensitized tumours is of the same order as that previously reported for directly attenuated tumour inocula treated at the same dose, at best about 50 per cent cures at 4200r. It seems probable that a similar mechanism operates in the two cases. Previous observations (Cohen and Cohen, 1954*b*, 1954*c*) suggested that the attenuated tissue had been rendered antigenic to the host, thus evoking some generalized systemic resistance mechanism, most probably a circulating iso-antibody. These subliminal immune reactions are apparently regularly associated with an increased radio-sensitivity of the tumour (Cohen and Cohen, 1954*a*). The results of the present experiment follow logically from, and lend strong support to this immunological hypothesis.

The tendency to late recurrence in many of the initially cured mice has not been previously observed in this laboratory, although a very large series of cured mice has now accumulated from many preceding experiments. It seems that the apparently cured tumours are not entirely eradicated by irradiation *in situ*, at least at this relatively ineffective dose level, but are subject to some continuing restraint or resistance on the part of the host. The phenomenon of prolonged tumour dormancy associated with host resistance, and recrudescence when this resistance fails, is also well-known in clinical cancer (Hadfield, 1954). The restraining process, perhaps the titre of the antibody, in common with most other immunological responses, tends to diminish with time, thus permitting late, slow-growing recurrences of tumours that were impalpable for some 3 months after treatment.

If the foregoing results are to be explained on an immunological hypothesis, then more effective immunizing procedures are likely to give still better responses. In line with past experience in immunology it may well be that animals bearing primarily unmodified tumours, to which they normally have little or no resistance, may require repeated " booster " inocula of attenuated tissue to maintain the remission. It is possible, too, that tumour tissue attenuated at doses higher than those used in the above experiments would be more effective, as the observed trend towards greater curability with larger attenuating doses was not fully explored.

### SUMMARY

C3H mice bearing isografts of the C3H mammary adenocarcinoma were inoculated with fragments of the same tumour that had been attenuated with varying doses of röntgen rays immediately prior to implantation. The originally established tumours were then irradiated *in situ* with 4200r, a dose at which the expectation of cure is normally under 0·1 per cent. In this experimental series, however, the cure rate at 4200r approached 50 per cent, indicating that the procedure had significantly increased the radio-sensitivity of the unmodified isogenic tumour. Optimal results were obtained when the attenuation dose was 5000r, the interval between implantation of the unmodified tumour and the attenuated inoculum was 3 to 21 days, and the interval between the attenuated inoculum and treatment *in situ* was 2 to 7 weeks.

A tendency to late recurrence was noted in some of these tumours apparently cured for 3 months after treatment.

It is considered that inoculation of the attenuated tumour fragment induces a subliminal and transient resistant state in the host, which enhances the radio-sensitivity of a previously established tumour.

The authors are deeply indebted to Dr. J. F. Murray, of the South African Institute for Medical Research, who has generously provided all the necessary facillities for the maintenance of the animals used in these experiments, and to Mrs. Shelley Jacobson for her expert assistance in this work.

### REFERENCES

Cohen, A. and Cohen, L.—(1953) *Brit. J. Cancer*, **7**, 231.—(1954*a*) *Ibid.*, **8**, 303.—(1954*b*) *Ibid.*, **8**, 313.—(1954*c*) *Ibid.*, **8**, 522.
Cramer, W.—(1932) *Sci. Rep. Cancer Res. Fd., Lond.*, **10**, 95.
Goldfeder, A.—(1940) *Radiology*, **35**, 210.
Hadfield, G.—(1954) *Brit. med. J.*, ii 607.